# Evaluation of low density polyethylene and nylon for delivery of synthetic mosquito attractants

**DOI:** 10.1186/1756-3305-5-202

**Published:** 2012-09-19

**Authors:** Wolfgang R Mukabana, Collins K Mweresa, Philemon Omusula, Benedict O Orindi, Renate C Smallegange, Joop JA van Loon, Willem Takken

**Affiliations:** 1 International Centre of Insect Physiology and Ecology, P.O. Box 30772-00100, Nairobi, Kenya; 2 School of Biological Sciences, University of Nairobi, P.O. Box 30197 GPO, Nairobi, Kenya; 3 Laboratory of Entomology, Wageningen University and Research Centre, P.O. Box 8031, 6700 EH, Wageningen, The Netherlands; 4 Enza Zaden R&D B.V, P.O. Box 7, 1600 AA, Enkhuizen, The Netherlands

**Keywords:** Mosquito, Trapping, Attractant, Odour release system

## Abstract

**Background:**

Synthetic odour baits present an unexploited potential for sampling, surveillance and control of malaria and other mosquito vectors. However, application of such baits is impeded by the unavailability of robust odour delivery devices that perform reliably under field conditions. In the present study the suitability of low density polyethylene (LDPE) and nylon strips for dispensing synthetic attractants of host-seeking *Anopheles gambiae* mosquitoes was evaluated*.*

**Methods:**

Baseline experiments assessed the numbers of *An. gambiae* mosquitoes caught in response to low density polyethylene (LDPE) sachets filled with attractants, attractant-treated nylon strips, control LDPE sachets, and control nylon strips placed in separate MM-X traps. Residual attraction of *An. gambiae* to attractant-treated nylon strips was determined subsequently. The effects of sheet thickness and surface area on numbers of mosquitoes caught in MM-X traps containing the synthetic kairomone blend dispensed from LDPE sachets and nylon strips were also evaluated. Various treatments were tested through randomized 4 × 4 Latin Square experimental designs under semi-field conditions in western Kenya.

**Results:**

Attractant-treated nylon strips collected 5.6 times more *An. gambiae* mosquitoes than LDPE sachets filled with the same attractants. The attractant-impregnated nylon strips were consistently more attractive (76.95%; n = 9,120) than sachets containing the same attractants (18.59%; n = 2,203), control nylon strips (2.17%; n = 257) and control LDPE sachets (2.29%; n = 271) up to 40 days post-treatment (P < 0.001). The higher catches of mosquitoes achieved with nylon strips were unrelated to differences in surface area between nylon strips and LDPE sachets. The proportion of mosquitoes trapped when individual components of the attractant were dispensed in LDPE sachets of optimized sheet thicknesses was significantly higher than when 0.03 mm-sachets were used (P < 0.001).

**Conclusion:**

Nylon strips continuously dispense synthetic mosquito attractants several weeks post treatment. This, added to the superior performance of nylon strips relative to LDPE material in dispensing synthetic mosquito attractants, opens up the opportunity for showcasing the effectiveness of odour-baited devices for sampling, surveillance and control of disease vectors.

## Background

The effectiveness of odour-baited tools for sampling, surveillance and control of insect vectors is strongly influenced by the selected odour delivery device [1,2]. Low density polyethylene (LDPE) materials have proved useful because odour baits are released at predictable rates and do not need to be replenished over prolonged periods of time [1,3]. However, these attributes may not guarantee maximal mosquito trap catches without prior optimization of sheet thickness and surface area [1,2,4]. Since LDPE sachets are prone to leakage, further searches for slow-release materials and techniques is warranted for the optimal release of odorants.

In a previous eight-day study we reported on the efficacy of nylon fabric (90% polyamide and 10% spandex) as a tool for dispensing odours [3]. A potent synthetic mosquito attractant namely Ifakara blend 1 (hereafter referred to as blend IB1) was used to evaluate open glass vials, LDPE and nylon as delivery tools. Nylon strips impregnated with blend IB1 attracted 5.83 and 1.78 times more *Anopheles gambiae* Giles *sensu stricto* (hereafter referred to as *An. gambiae*) mosquitoes than solutions of attractants dispensed from glass vials and LDPE sachets, respectively [3]. However, in the case of nylon strips each chemical component of the attractant was applied at its optimal concentration whereas such optimization had not been implemented in advance for LDPE sachets.

In this study we re-evaluated the suitability of nylon versus LDPE as materials for dispensing synthetic mosquito attractants. We pursued four specific aims i.e. (i) comparison of nylon strips and LDPE sachets as materials for releasing synthetic mosquito attractants, (ii) assessment of the residual activity of attractant-baited nylon strips and LDPE sachets on host-seeking mosquitoes, (iii) determination of the effect of LDPE sheet thickness on attraction of mosquitoes to synthetic attractants, and (iv) comparison of surface area effects on attraction of mosquitoes to attractants administered through nylon strips versus LDPE sachets.

## Methods

The study was carried out at the Thomas Odhiambo Campus of the International Centre of Insect Physiology and Ecology (*icipe*) located near Mbita Point Township in western Kenya between April 2010 and January 2011.

### Mosquitoes

The Mbita strain of *An. gambiae* was used for all experiments. For maintenance of this strain, mosquito eggs were placed in plastic trays containing filtered water from Lake Victoria. Larvae were fed on Tetramin® baby fish food three times per day. Pupae were collected daily, put in clean cups half-filled with filtered lake water and then placed in mesh-covered cages (30 × 30 × 30 cm). Emerging adult mosquitoes were fed on 6% glucose solution.

### General procedures

The experiments were conducted under semi-field conditions in a screen-walled greenhouse measuring 11 m × 7 m × 2.8 m, with the roof apex standing 3.4 m high. Four treatments including two negative controls were evaluated in each experimental run. A total of 200 adult female mosquitoes aged 3–5 days old were utilized for individual bioassays conducted between 20:00 and 06:30 h. The mosquitoes were starved for 8 h with no prior access to blood meals. Only water presented on cotton towels on top of mosquito holding cups was provided. Mosquitoes attracted to each treatment were sampled using MM-X traps (American Biophysics, North Kingstown, RI, USA). The nylon strips and LDPE sachets were suspended inside the plume tubes of separate traps where a fan blew air over them to expel the attractant plume as indicated in our previous study [3]. Latex gloves were worn when hanging odour dispensers in the traps to avoid contamination.

Trap positions were rotated to minimise positional effects. The traps were placed 1 m away from the edges of the greenhouse [4-6]. Each trap was marked and used for one specific treatment throughout the experiments. The number of mosquitoes collected per trap was counted and used both as an estimate for the attractiveness of the baits and an indicator for the suitability of dispensing materials. Each morning the traps were cleaned using 70% methanol solution. Mosquitoes that were not trapped were recaptured from the green house using manual aspirators and killed. Temperature and relative humidity in the greenhouse were recorded using data loggers (Tinytag®). Whereas all experiments were conducted for 12 nights, responses of mosquitoes to residual release from attractant-treated nylon strips were evaluated for 40 nights and repeated three times.

### Response of mosquitoes to attractant-treated nylon strips versus LDPE sachets

A 4 × 4 Latin square experimental design was conducted incorporating LDPE sachets filled with IB1, IBI-treated nylon strips, LDPE sachets filled with water (hereafter termed control LDPE sachets) and water-treated nylon strips (hereafter termed control nylon strips) as treatments. Sheet thicknesses of LDPE sachets each measuring 2.5 cm × 2.5 cm (surface area 12.5 cm^2^) were optimized for individual chemical components of blend IB1 [7]. These were 0.2 mm (distilled water, propionic, butanoic, pentanoic, and 3-methylbutanoic acid), 0.1 mm (heptanoic and octanoic acid), 0.05 mm (lactic acid) and 0.03 mm (tetradecanoic acid and ammonia solution). Depending on treatment, LDPE sachets were filled with either 1 ml of the attractant compound or solvent. Individual nylon strips measuring 26.5 cm × 1 cm (surface area 53 cm^2^) were separately soaked in 1 ml of each of the chemical constituents of blend IB1 at their optimal concentrations [3,7]. The strips were air-dried at room temperature for 5 h before the start of experiments. Whereas attractant-treated nylon strips were freshly prepared each day, LDPE sachets filled with IB1 were re-used throughout the 12 days of the study and replaced upon leakage or depletion of individual components. Carbon dioxide, produced from 250 g of sucrose dissolved in 2 l of tap water containing 17.5 g of yeast [3,5,8] was supplied through silicon gas tubing at a flow rate of approximately 63 ml/min into traps baited with IB1-treated nylon strips or LDPE sachets filled with IB1 only and not with control nylon or LDPE sachets. Individual LDPE sachets containing chemicals were weighed before and after each experiment to determine how much of the individual components of the blend had been released. Control LDPE sachets and LDPE sachets filled with IB1 were stored in the refrigerator at 4 ^0^C between experimental runs.

### Residual activity of attractant-treated nylon strips on host seeking mosquitoes

In our previous study we noted the potential disadvantage of nylon strips i.e. that they tend to dry up quickly so no more active ingredient may be available following long hours of trap operation [3]. We designed experiments aimed at addressing this shortcoming. A 4 × 4 Latin square experimental design was used to evaluate residual attraction of *An. gambiae* to IB1-treated nylon strips and LDPE sachets filled with IB1. The four treatments included (i) LDPE sachets filled with IB1, (ii) IB1-treated nylon strips, (iii) control LDPE sachets and (iv), control nylon strips. The number of mosquitoes attracted to each treatment over a period of 40 nights was recorded daily and proportions trapped were calculated. The experiment was replicated three times. Analysis of data revealed no need to prepare fresh nylon strips daily. Thus, nylon strips were re-used in subsequent experiments. Whereas control LDPE sachets and IB1-filled LDPE sachets were also re-used, individual sachets were replenished upon depletion of contents. Sachets containing butanoic, pentanoic, 3-methylbutanoic, heptanoic and octanoic acid were replaced after every 10–14 nights.

### Sheet thickness of LDPE sachets containing attractants and its effect on attraction of *An. gambiae*

Direct exposure of IB1-treated nylon to environmental conditions may have led to higher release rates of attractant volatiles resulting in more mosquitoes being attracted relative to LDPE sachets of optimal sheet thicknesses containing the same attractants. We hypothesized that increasing release rates for all components in the blend using IB1-filled LDPE sachets of 0.03 mm sheet thickness for all components in the blend (hereafter indicated as 0.03 mm-LDPE or 0.03 mm-sachet) could enhance numbers of mosquitoes attracted. A sheet thickness of 0.03 mm was selected, because it was the thinnest available LDPE material and had been used in our previous investigations [7]. This hypothesis was tested by comparing *An. gambiae* mosquito capture rates with sachets of variable thickness versus 0.03 mm sachets. The sachets were weighed daily before and after each experiment to verify differences in volatile release rates. The carbon dioxide component of the blend was delivered separately through silicon tubing. A randomised 4 × 4 Latin square experimental design was adopted. The treatments included (a) LDPE sachets with optimized sheet thicknesses for all components of IB1, (b) each component of IB1 dispensed in LDPE sachets of 0.03 mm sheet thickness, (c) control LDPE sachets with optimal sheet thicknesses for all components of IB1, and (d) control LDPE sachets with 0.03 mm sheet thickness.

### Response of mosquitoes to attractants applied on nylon versus 0.03 mm LDPE sachets

In addition to investigating the effect of volatile release rates on mosquito behaviour, we compared numbers of *An. gambiae* mosquitoes attracted to IB1-filled in LDPE sachets of uniform sheet thickness (0.03 mm) or applied on nylon strips. The following treatments were tested (a) IB1-treated nylon strips, (b) each component of IB1 dispensed in 0.03 mm-LDPE sachets, (c) control nylon strips and (d) control 0.03 mm-LDPE sachets. A randomised 4 × 4 Latin square experimental design was adopted. The sachets and nylon strips had surface areas of 12.5 cm^2^ and 53 cm^2^, respectively.

### Effects of dispenser surface area on attraction of mosquitoes

As higher mosquito catches associated with IB1-treated nylon strips could not be explained by the strips being freshly treated prior to each experiment, we tested whether variations in mosquito catches were due to differences in surface area. The LDPE sachets and nylon strips used in previous experiments of this study had surface areas of 12.5 cm^2^ and 53 cm^2^, respectively. Thus, the strips released odorants over a larger surface area than the LDPE sachets. We designed two sets of 4 × 4 Latin square experiments to test whether the larger surface area of nylon strips was responsible for the higher mosquito catches. The four treatments included (a) IB1-treated nylon strips, (b) LDPE sachets filled with IB1, (c) control nylon strips and (d) control LDPE sachets. Enlarged LDPE sachets were similarly filled with one ml of attractant or solvent. In the first set of experiments, surface areas of control and attractant-filled LDPE sachets were enlarged (2.5 cm wide × 10.6 cm long × 2 sides of the sachet) to equal the surface area of nylon strips. In the second set of experiments, a piece of absorbent material (nylon strip) was placed inside enlarged (53 cm^2^) control and attractant-filled LDPE sachets to ensure that blend IB1 was evenly spread over the entire inner surface of the sachets. Each set of experiments was replicated 12 times. All other experimental procedures were similar to those described in previous sections.

### Data analysis

The relative efficacy of each treatment was defined as a percentage of female mosquitoes caught in the traps containing either of the two release materials impregnated or filled with synthetic attractants or solvent. In order to investigate the effect of residual activity of attractant-treated materials on capture rates, we used the baseline-category logit model [9]. The nominal response variable was defined as the attractant type with four categories: IB1-containing LDPE sachets, IB1-treated nylon strips, control nylon strips, and control LDPE sachets with day and trap position as covariates. We estimated the odds that mosquitoes chose other attractants instead of IB1-treated nylon strips over time, while adjusting for trap-position. The Mann Whitney-*U* test was used to estimate the effect of sheet thickness of LDPE sachets on release rates of IB1 components except carbon dioxide. To investigate the effect of surface area and sheet thickness on the release material on mosquito catches, we fitted a Poisson regression model controlling for trap position. The analyses were performed using SAS v9.2 (SAS Institute Inc.) with tests performed at 5% level.

## Results

### Response of mosquitoes to attractant-treated nylon strips versus LDPE sachets

**Figure 1:**
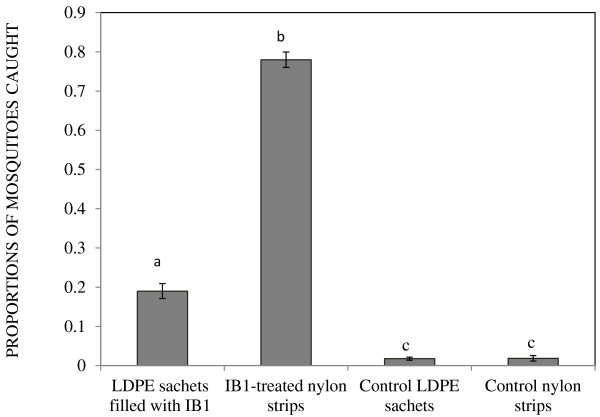
**Proportions of mosquitoes caught in MM-X traps containing IB1-treated nylon strips, LDPE sachets filled with blend IB1, control nylon strips and control LDPE sachets.** Mean mosquito catches represented by bars with different letters differ significantly (P < 0.05). Error bars represent the standard error of the mean proportion of mosquito catches.

The 12-day period over which experiments were conducted was characterized by a mean temperature and relative humidity of 22.18 ± 0.08^0^C and 86.15 ± 1.56%, respectively, within the screen-walled greenhouse. Out of 2,400 female *An. gambiae* mosquitoes released, 51.88% (n = 1,235) were caught in the four treatment traps. Of these catches, 77.73%, 18.62%, 1.78% and 1.86% were trapped by IB1-treated nylon strips, LDPE sachets filled with IB1, control nylon strips and control LDPE sachets, respectively (Figure 1). Baseline-category logit model results revealed that IB1-impregnated nylon strips attracted, on average, 5.6 times more mosquitoes than LDPE sachets filled with IB1 (P < 0.001). Whereas there was no significant difference in the proportion of mosquitoes attracted to control nylon strips and control LDPE sachets (P = 0.436), these treatments attracted significantly fewer mosquitoes than nylon and LDPE sachets containing blend IB1 (P < 0.001). Day effect was not significant (P = 0.056), and was therefore excluded from the final model. However, trap position was an important determinant of mosquito catches (P < 0.001). These experiments provided baseline information for subsequent investigations conducted during the study.

### Residual activity of attractant-treated nylon strips on host-seeking mosquitoes

**Figure 2:**
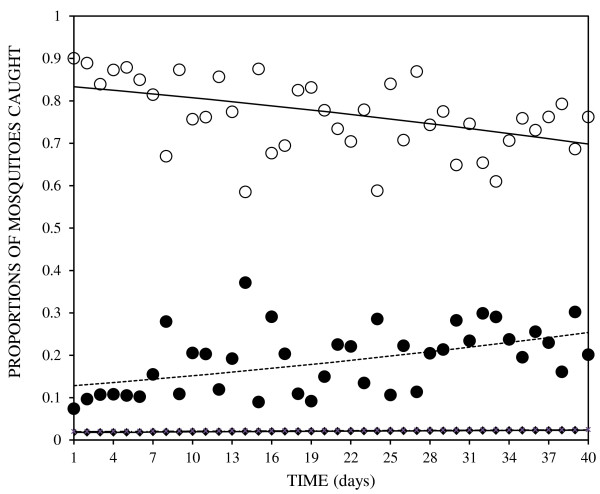
**Proportions of mosquitoes caught in traps containing IB1-treated nylon strips (―), LDPE sachets filled with blend IB1 (−−--), control nylon strips (—♦--) and control LDPE sachets (− × − × −) over time.** Lines and symbols representing mosquito catches due to control nylon strips and control LDPE sachets are superimposed over each other. Open (IB1-treated nylon strips) and closed circles (LDPE sachets filled with IB1) represent observed values. Lines represent the Baseline-category logit model fit showing trends of proportions of mosquitoes attracted over time.

A total of 11,851 (49.38%) mosquitoes were attracted and collected over 120 nights (i.e. three replicates of 40 days each). The proportions of mosquitoes caught over time differed among treatments (P < 0.001) (Figure 2). Attractant-treated nylon strips repeatedly trapped the highest proportion of mosquitoes without re-applying the attractant blend up to 40 days post-treatment. During this period the treated nylon strips, LDPE sachets filled with IB1, control nylon strips and control LDPE sachets attracted 76.95% (n = 9,120), 18.59% (n = 2,203), 2.17% (n = 257) and 2.29% (n = 271) of the mosquitoes, respectively. There was also a significant increase over time in the proportion of mosquitoes choosing LDPE sachets filled with IB1 (P < 0.001), but not for control nylon strips (P = 0.051) and control LDPE sachets (P = 0.071). In contrast, the numbers of mosquitoes attracted to IB1-impregnated nylon strips decreased considerably over time (P < 0.002). However, they were consistently preferred to LDPE sachets filled with IB1 (Figure 2).

### Sheet thickness of LDPE sachets containing attractants and its effect on attraction of *An. gambiae*

Here LDPE sachets with sheet thickness optimized [7] or kept uniform (0.03 mm) for each chemical constituent of the attractant were evaluated. Out of 2,400 mosquitoes released, 51.17% were trapped (Table 1). Whereas trap position was not a significant factor (P = 0.183), attraction of mosquitoes to different traps was influenced by LDPE sheet thickness (P < 0.001). Delivery of attractant components through sachets with optimized sheet thicknesses resulted in a significant increase in mosquito catches as opposed to uniform 0.03 mm-sachets (P < 0.001). There was no difference in mosquito catches between both types of control LDPE sachets (P = 0.111).

**Table 1:** **Effect of polyethylene sheet thickness on attraction of *An. gambiae* to attractant baited sachets**

**Treatment**	**N**	**Number of mosquitoes trapped**	
		**n**	**Mean ± S.E**
Blend IB1 in sachets with optimal sheet thickness	12	633	52.75 ± 4.46^a^
Blend IB1 in 0.03 mm-sachets	12	495	41.25 ± 5.2^b^
Control sachets (optimal sheet thickness)	12	58	4.83 ± 0.9^c^
Control 0.03 mm-sachets	12	42	3.50 ± 0.8^c^

N refers to the number of replicates and n to the total number of mosquitoes trapped. Mean (±S.E) numbers of mosquitoes trapped with different letter superscripts differ significantly (P < 0.05).

**Figure 3:**
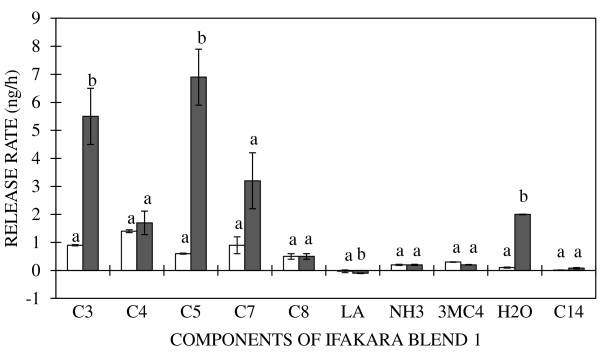
**Effect of LDPE sheet thickness on release rates of chemical constituents contained in the mosquito attractant Ifakara blend 1 (IB1).** Release rates from sachets, the sheet thickness of which had been optimised for all chemicals components of the blend (open bars) or kept uniform (0.03 mm-sheets) for all the chemical constituents (shaded bars), are shown. The optimised LDPE sheet thicknesses were 0.2 mm [distilled water (H_2_0), propionic (C3), butanoic (C4), pentanoic (C5), and 3-methylbutanoic acid (3MC4)], 0.1 mm [heptanoic (C7) and octanoic acid (C8)], 0.05 mm [lactic acid (LA)] and 0.03 mm [tetradecanoic acid (C14) and ammonia solution (NH_3_)]. Odour release rates represented by bars with different letters differ significantly (P < 0.05). Error bars represent the standard error of the mean odour release rates measured in ng/h.

The effect of porosity due to differences in sheet thickness of LDPE sachets on release rates of various chemicals emitted from blend IB1 was also investigated. Mann Whitney-U tests indicated that sheet thickness had a significant effect on the release rates of propionic acid, pentanoic acid, heptanoic acid, distilled water and lactic acid (P = 0.04, 0.03, 0.02, 0.01 and 0.02, respectively). However, release rates of butanoic acid, 3-methylbutanoic acid, octanoic acid, tetradecanoic acid, and ammonia were not dependent on sheet thickness of LDPE sachets (P = 0.722, 0.97, 0.30, 0.23, and 0.87, respectively) (Figure 3).

### Response of mosquitoes to attractant-treated nylon versus thin-sheeted polyethylene sachets

**Figure 4:**
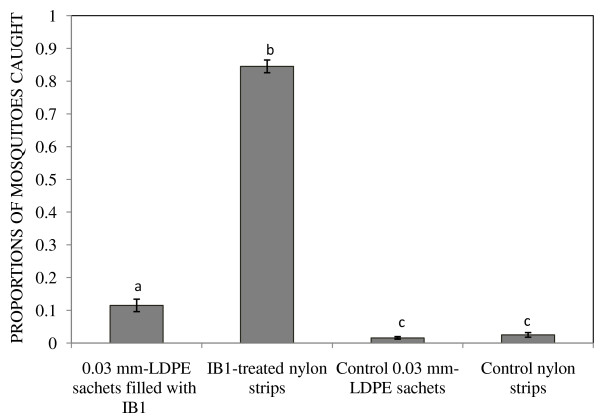
**Proportions of mosquitoes caught by traps containing IB1-treated nylon strips, 0.03 mm-LDPE sachets filled with blend IB1, control 0.03 mm-LDPE sachets and control nylon strips.** Mosquito catches represented by bars with different letters differ significantly (P < 0.05). Error bars represent the standard error of the mean proportion of mosquito catches.

Additional studies confirmed that mosquitoes preferred attractant-treated nylon strips compared to attractants contained in 0.03 mm-LDPE sachets (P < 0.001). Overall, 49.63% (n = 1191) of released mosquitoes were recaptured. Of these, 84.50%, 11.07%, 2.26%, and 1.68% were found in traps baited with attractant-treated nylon strips, LDPE sachets (0.03 mm) filled with IB1, control nylon strips and control LDPE sachets (0.03 mm), respectively (Figure 4). The numbers of mosquitoes caught by control strips and sachets were not significantly different (P = 0.309).

### Effects of dispenser surface area on attraction of mosquitoes

The LDPE sachets and nylon strips used to dispense blend IB1 in preceding experiments of this study had total surface areas of 12.5 cm^2^ and 53 cm^2^, respectively. Follow-up experiments were conducted in which LDPE sachets were enlarged (2.5 cm × 10.6 cm × 2) to equal the surface area of the nylon strips. Attractant-treated nylon strips caught significantly more mosquitoes than attractants contained in enlarged LDPE sachets with (P < 0.001) and without an inner lining of absorbent material (P < 0.001) (Table 2). Thus, higher attraction of mosquitoes to IB1-treated nylon strips was not neutralized by equalized surface area or uniform spread of attractants over the inner surface area of LDPE sachets. Mosquito responses to traps containing control nylon strips versus control LDPE sachets with or without the absorbent nylon material were not different ((P = 0.173 and P = 0.556, respectively). Position had a significant effect on trap catches (P < 0 .001 in both cases).

**Table 2:** Behavioural responses of mosquitoes towards attractant treated polyethylene sachets lined with nylon versus nylon strips treated with a similar attractant

**Treatment**	**N**	**Proportions of mosquitoes caught**			
		**LDPE (no absorbent)**		**LDPE (with absorbent)**	
		**n**	**Mean ± S.E**	**n**	**Mean ± S.E**
IB1-treated nylon strips	12	968	78.20 ± 2.57^a^	1066	86.02 ± 2.7^a^
IB1-filled LDPE sachets	12	282	22.78 ± 1.37^b^	320	25.82 ± 1.45^b^
Control nylon strips	12	28	2.35 ± 0.44^c^	26	2.10 ± 0.41^c^
Control LDPE sachets	12	35	2.73 ± 0.46^c^	17	1.37 ± 0.33^c^

N refers to the number of replicates and n to the total number of mosquitoes trapped. Mean (±S.E) numbers of mosquitoes trapped with different letter superscripts differ significantly (P < 0.05).

## Discussion

This study demonstrates that nylon strips can act as a sustainable matrix for dispensing synthetic attractants of host-seeking *An. gambiae* mosquitoes, performing much better than low density polyethylene (LDPE) sachets. It was remarkable that attractant-treated nylon strips continued to attract mosquitoes without re-application and remained consistently more attractive than LDPE sachets filled with the same attractants over a period of 40 nights post-treatment. The higher catches of mosquitoes associated with nylon strips were apparently not due to smaller surface area, uneven spread of the attractant on inner surfaces or LDPE sheet thickness.

The baseline experiments reported herein confirm findings of our previous studies in which nylon strips were found to provide a better release matrix for delivering synthetic attractants of host-seeking *An. gambiae* mosquitoes than did LDPE sachets or open glass vials [3]. LDPE and nylon differ in physico-chemical characteristics such as porosity and chemical binding affinity that may explain the observed differences in mosquito catches through their effects on the release rate of odorant volatiles [1,10,11]. Although the use of LDPE sachets allows the adjustment of attractant release rates, release rates from nylon have yet to be determined e.g. through headspace sampling at the trap outlet.

That IB1-treated nylon strips remained consistently more attractive to host-seeking *An. gambiae* mosquitoes than LDPE sachets filled with the same attractants for a period of up to 40 days post-treatment is definitive proof of inherent residual activity. This finding corroborates that of related studies where nylon stockings impregnated with human emanations remained attractive to *An. gambiae* mosquitoes for several weeks [12-14]. Blend IB1 impregnated on nylon strips may have been subject to bacterial degradation over the prolonged experimental time. This may have resulted in the release of additional components than were originally present on the nylon strips [15-17]. However, the present study did not investigate the presence of microbes or additional attractant compounds on aging IB1-treated nylon strips.

The current study shows that, attractant-treated nylon strips can be re-used for at least 40 consecutive days as baits for host-seeking *An. gambiae* mosquitoes, thereby reducing costs of odorants and nylon strips, time and labour used to prepare fresh baits. These attributes are consistent with those associated with the long-lasting fabric materials impregnated with mosquito repellents or insecticides [18,19]. The availability of long-lasting mosquito-attractant fabrics is interesting as these can potentially be combined with mosquito pathogens such as entomopathogenic fungi or bacteria [20]. Thus, a cheap and effective tool for intercepting and eliminating host-seeking mosquitoes can be exploited for vector-borne disease control. However, further testing is needed to examine the maximal duration of residual activity of the attractant-treated strips.

Contrary to our expectations, LDPE sachets optimized for release rates and surface area caught fewer mosquitoes than nylon strips. The release rate of some compounds (propanoic, pentanoic, heptanoic, lactic acid and water) was significantly increased when uniformly thinner-sheeted sachets were utilized. Because sheet thickness of LDPE sachets is a determinant of volatile release rate the composition of the volatile blend released may have changed so as to negatively affect attractiveness to *An. gambiae* mosquitoes [1,21]**.** We conclude that, blend ratio and concentration affects orientation and capture rates of insect vectors with odour-baited systems [22,23].

Although LDPE sachets have been effectively used to release attractants for tsetse flies and other insect pests [1,2], they attracted fewer mosquitoes compared to nylon strips when both were treated or filled with the same blend of attractants. This could be explained by differences in optimized sheet thicknesses of LDPE sachets and physical and chemical characteristics of the odorants used for attraction of tsetse flies versus those used for mosquitoes [24]. Moreover, trap designs used for collection of both insect vectors were also different [3,25,26]. Delivery of synthetic attractant components through sachets with standardized sheet thickness and surface area have demonstrated consistent mosquito catches under laboratory and semi-field conditions [17].Whereas nylon strips were associated with higher mosquito catches, we currently lack information on the release rates of the odorants dispensed. Accurate release rates have been established for odorants delivered through LDPE sachets [4], and such chemical measurements should also be done for nylon, as this allows for a direct comparison of the active aerial odorant concentration that host-seeking mosquitoes encounter.

## Conclusion

This study demonstrates that nylon strips present a potent and sustainable release material for dispensing synthetic mosquito attractants. Apparently, attractant-treated nylon strips can be used over prolonged time without re-applying the attractant blend. Treatment of nylon surfaces with attractants presents an opportunity for use in long-lasting odour-baited devices for sampling, surveillance and control of disease vectors.

## Competing interests

The authors declare that they have no competing interests.

## Authors’ contributions

WRM, CKM, WT, and RCS designed the study; CKM, and PO conducted the research; BO and WRM analysed the data; CKM, WRM, RCS, WT and JJAvL wrote the paper. All authors read and approved the final manuscript.
